# Supplementary material for: Lignin–Cobalt Nano-Enabled Poly(pseudo)rotaxane Supramolecular Hydrogel for Treating Chronic Wounds
Source: Pharmaceutics. 2023 Jun 13;15(6):1717. doi: 10.3390/pharmaceutics15061717 (PMC10301185; doi:10.3390/pharmaceutics15061717)
Supplement: Supplementary file 1 [file pharmaceutics-15-01717-s001.zip › pharmaceutics-2349754-supplementary.pdf]

pharmaceutics

MDPI

Supplementary Materials: Lignin–Cobalt Nano-Enabled Poly(pseudo)rotaxane Supramolecular Hydrogel for Treating Chronic Wounds

Giulia Crivello, Giuliana Orlandini, Angela Gala Morena, Alessandro Torchio, Clara Mattu, Monica Boffito, Tzanko Tzanov and Gianluca Ciardelli

Supplementary information:

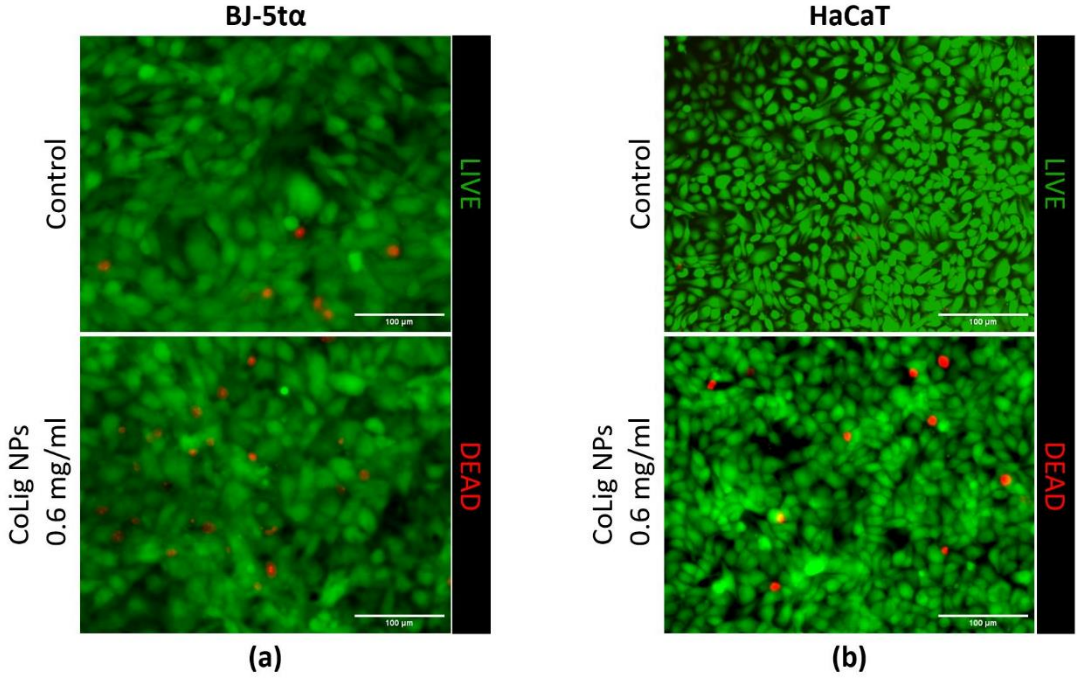

Figure S1. Live/dead assay for human fibroblast (BJ-5ta) and keratinocytes (HaCaT) treated with CoLig NPs at a cytocompatible concentration of 0.6 mg/ml.

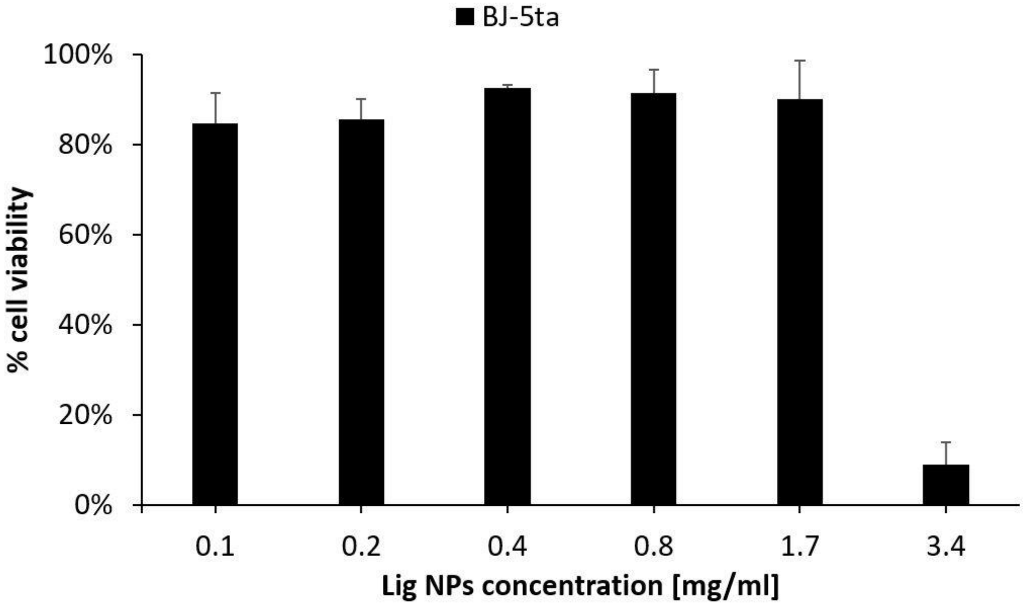

Figure S2. Cytotoxicity of Lig NPs against human fibroblast (BJ-5ta).

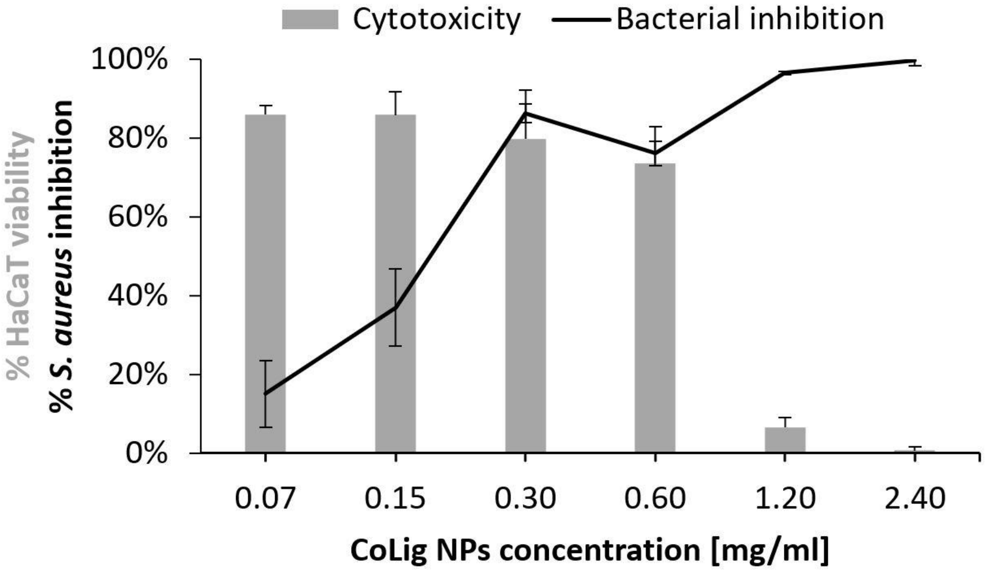

Figure S3. Comparison between cytotoxicity toward HaCaT and *S. aureus*-inhibitory ability of CoLig NPs.

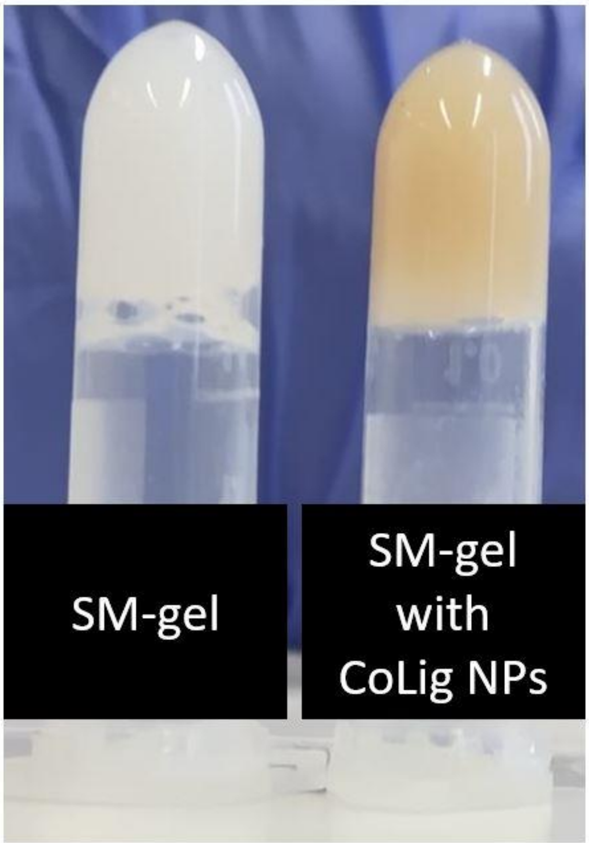

Figure S4. SM-gel with and without CoLig NPs. The result of the CoLig NPs integration is a homogeneous hydrogel with brown coloration related to the presence of lignin.

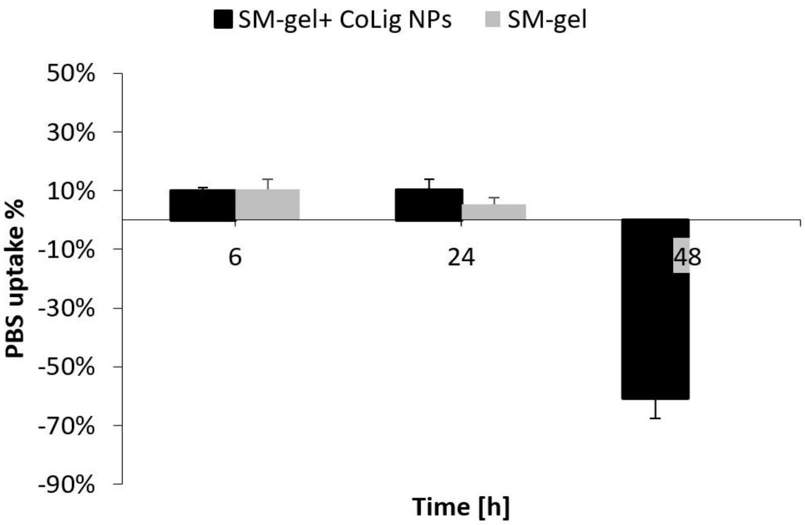

Figure S5. PBS uptake % of SM-gel with and without CoLig NPs.

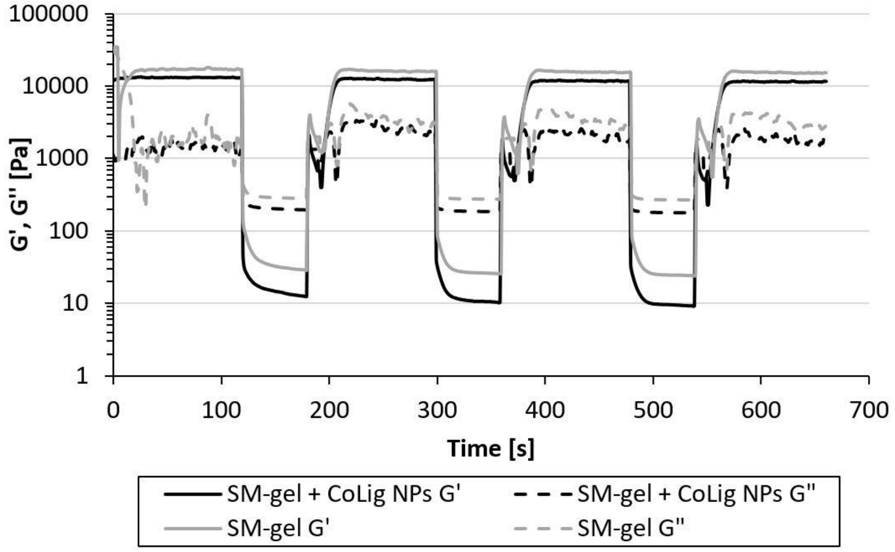

Figure S6. Self-healing strain tests for the SM-gel with and without CoLig NPs.

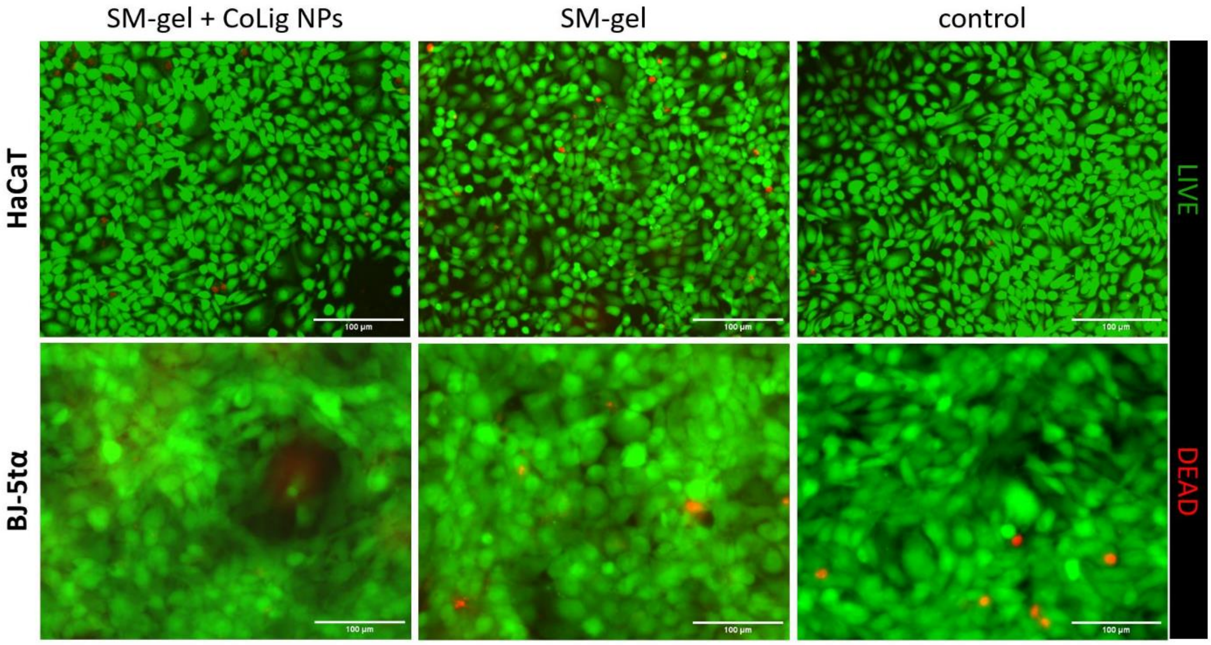

Figure S7. Live/dead assay for human fibroblast (BJ-5ta) and keratinocytes (HaCaT) treated with SM-gel with and without CoLig NPs.
